# Mitochondrial D-loop Sequence Variability in Three Native Insular Griffon Vulture (*Gyps fulvus*) Populations from the Mediterranean Basin

**DOI:** 10.1155/2019/2073919

**Published:** 2019-11-18

**Authors:** Paolo Mereu, Monica Pirastru, Valentina Satta, Gian Nicola Frongia, Nicolaos Kassinis, Minas Papadopoulos, Eleftherios Hadjisterkotis, Stavros Xirouchakis, Laura Manca, Salvatore Naitana, Giovanni Giuseppe Leoni

**Affiliations:** ^1^Department of Biomedical Sciences, University of Sassari, Sassari, Italy; ^2^Department of Veterinary Medicine, University of Sassari, Sassari, Italy; ^3^Game and Fauna Service, Ministry of the Interior, Nicosia, Cyprus; ^4^Department of Forests, Ministry of Agriculture, Rural Development and Environment, Nicosia, Cyprus; ^5^Agricultural Research Institute, Ministry of Agriculture, Rural Development and Environment, Nicosia, Cyprus; ^6^Natural History Museum of Crete, University of Crete, Crete, Greece

## Abstract

The islands of Sardinia, Crete, and Cyprus are hosting the last native insular griffon populations in the Mediterranean basin. Their states have been evaluated from “vulnerable” to “critically endangered”. The sequence analysis of molecular markers, particularly the mtDNA D-loop region, provides useful information in studying the evolution of closely related taxa and the conservation of endangered species. Therefore, a study of D-loop region sequence was carried out to estimate the genetic diversity and phylogenetic relationship within and among these three populations. Among 84 griffon specimens (44 Sardinian, 33 Cretan, and 7 Cypriot), we detected four haplotypes including a novel haplotype (HPT-D) that was exclusively found in the Cretan population with a frequency of 6.1%. When considered as a unique population, haplotype diversity (Hd) and nucleotide diversity (*π*) were high at 0.474 and 0.00176, respectively. A similar level of Hd and *π* was found in Sardinian and Cretan populations, both showing three haplotypes. The different haplotype frequencies and exclusivity detected were in accordance with the limited matrilineal gene flow (*F_ST_* = 0.07097), probably related to the species reluctance to fly over sea masses. The genetic variability we observe today would therefore be the result of an evolutionary process strongly influenced by isolation leading to the appearance of island variants which deserve to be protected. Furthermore, since nesting sites and food availability are essential elements for colony settlement, we may infer that the island's colonization began when the first domestic animals were transferred by humans during the Neolithic. In conclusion, our research presents a first contribution to the genetic characterization of the griffon vulture populations in the Mediterranean islands of Sardinia, Crete and Cyprus and lays the foundation for conservation and restocking programs.

## 1. Introduction

The European griffon (Accipitridae; Aegypetinae; *Gyps*; *G. fulvus*) is a vulture species widely distributed from the Mediterranean Basin in the west to the Indian subcontinent in the east. The species breeding range extends over Europe to the Middle East and North Africa including some island populations in the Mediterranean i.e., Majorca, Sardinia, Crete, Cyprus, and the Croatian islets of Cres, Krk, Prvić, and Plavnik. The European population is currently estimated at 32,400-34,400 pairs with the bulk of it residing on the Iberian Peninsula (BirdLife International 2017).

Griffons breed colonially on cliffs and rocky outcrops and feed communally almost exclusively on the carrion of medium-sized livestock and wildlife animals [1–3]. Due to their population size and feeding habits, they play a crucial role in Mediterranean ecosystems. By recycling carrion biomass, they limit the production of toxic molecules and CO_2_ related to carrion burning and consequently alleviate significantly the effects of global warming. In the meantime, they break the chain of infectious diseases of carcasses rotting in the field [4–6]. Broad wings and thus low wing-loading and low aspect ratio enable griffons to exploit effectively thermals, but, in any case, soaring flight that depends on thermals determines to a large extent their dispersal ability and migratory behaviour. Normally, they concentrate in specific locations such as peninsulas and land straits (e.g. Gibraltar, Suez) avoiding to fly over water. In fact, griffons are quite reluctant to cross even short distances over the sea (i.e. <25 km), and prefer to fly over land [7, 8]. The poor trend to cross short sea traits would be attributed to the absence of ascending thermal air currents in the sea associated to the risk of fatigue deriving by an improvement of flapping flight and the possibility of drifting off course due to crosswinds [9]. The Strait of Gibraltar wideness would represent the maximum limit beyond which sea crossing by griffon vulture is possible [8]. Therefore, in the case of islands, the greater their distance from the mainland, the more unlikely their colonization by the species. For the same reason, when some individuals occasionally reach an island, it would be difficult for them to come back onto the mainland. The islands' colonization can be due to exceptional meteorological events such as in the case of Majorca [10], or it can be traced back to the exploration of new areas by young individuals which show a natal dispersion [11], in contrast to adult griffons that are faithful to their breeding range [12, 13]. However, in comparison to adults, juvenile griffons exhibit inferior thermal soaring performance in their first two months after fledging due to the inexperience in thermal selection and centring, and interthermal gliding soaring [14]. This fact suggests that juveniles make worse choices compared to adults, but with a higher potential for colonizing new ecosystems like islands.

Currently, the Mediterranean islands of Majorca, Sardinia, Croatian islets of the Kvarner Archipelago, Crete, and Cyprus are hosting insular griffon populations. However, apart from the Adriatic colonies that are close to the Croatian coast (1–3.5 km), the rest of them would have had a very low level of exchange with continental griffon populations given their distance from mainland Europe. Because of this geographical isolation and difficulties to cross sea stretches, these griffon populations may have maintained distinctive genetic characteristics compared to the mainland colonies. Le Gouar et al. [15] investigated, by means of microsatellite markers, the diversity, structure, and connection of native, captive founding, and reintroduced griffon populations from Spanish, French, and Israeli mainland colonies. Results suggested a panmictic griffon population in the Mediterranean region with a low genetic differentiation [15]. An exception was represented by the Croatian insular colony which was found significantly different from all others. The authors suggested that extinctions of geographically intermediate populations may have recently isolated Croatia from others colonies. Another study based on microsatellite analysis [16] confirmed the absence of population genetic structure among griffon vulture from Spain and Israel according to the past existence of high dispersal rates among griffon populations from Mediterranean region. Moreover, Arshad et al. [16] reported similar findings in samples from Cyprus population. A recent study investigating the mitochondrial DNA (mtDNA) sequence variability in the Sardinian griffon vulture population detected a mild level of genetic diversity as confirmed by the discovery of three haplotypes on 66 individuals [17]. This result acquires value taking into account the low genetic diversity found in previous studies investigating the mtDNA sequence variability within the entire *Gyps* genus [18, 19], and is consistent with Le Gouar et al. [15] which pointed out a higher genetic diversity level in *G. fulvus* than those detected in other vultures species such as *G. barbatus* and *Neophron percnopterus*.

The aim of the present study was to assess the genetic diversity and phylogenetic relationship within and among native griffon vulture populations hosted on the Mediterranean islands of Sardinia, Crete, and Cyprus by analysing the D-loop region. The species in Crete numbers 250–300 egg-laying pairs or an estimated total population of ca. 1000 individuals and has been evaluated as “vulnerable” [20]. In Cyprus and Sardinia the griffon vulture has been up-listed to “critically endangered”, the population census in 2004 provided an estimate of 8 egg-laying pairs nesting in the South-West of Cyprus and 42 egg-laying pairs nesting in North-West of Sardinia [21, 22].

## 2. Material and Methods

### 2.1. Sample Collection

**Figure 1:**
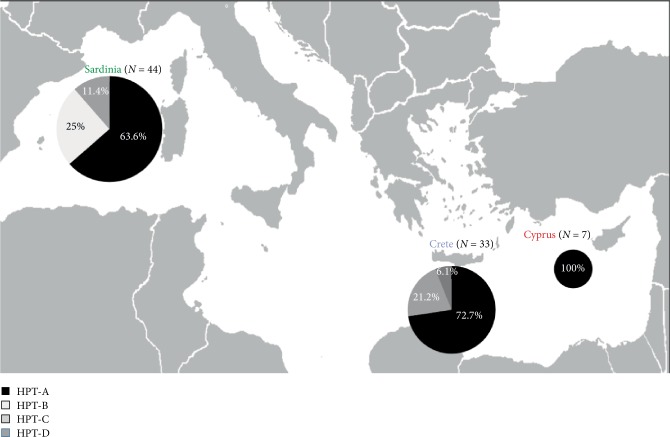
Geographical distribution and genetic structuring based on mtDNA haplotype analysis of griffon vulture in the Mediterranean islands of Sardinia, Crete, and Cyprus. The area of each circle is proportional to sample size. *N* = number of collected specimens.

Griffon vulture DNA samples were collected during research conducted by the Veterinary Medicine Department of the Sassari University (Italy), while operating in partnership with the Natural History Museum of the University of Crete (Greece) and the Ministry of the Interior of Cyprus (Cyprus). Overall, 84 blood and feather samples of living autochthonous individuals from Sardinia (*n* = 44), Crete (*n* = 33) and Cyprus (*n* = 7) were collected between January 2016 and December 2018 (Table 1, Figure 1).

Blood samples were collected with EDTA as anticoagulant by venal puncture using sterile equipment and with respect to the animal welfare. The Ethics Committee of Sassari University, Italy, reviewed all proposed procedures, including animal care and used protocols, prior to approval. No griffons have been sacrificed specifically for the present study.

### 2.2. Molecular Analysis

Specific mtDNA primers (GbCR1.L – GbCR2.H) designed by Johnson et al. [18] were used to amplify approximately 400 base pairs (bp) from the 5′ end of the mtDNA control region which has been found to contains all of the nucleotide variability discriminating the *G. fulvus* haplotypes detected within the Sardinian griffon population [17].

Genomic DNA isolation from blood samples and feathers was performed by using the NucleoSpin Blood kit (MACHEREY-NAGEL) and the NucleoSpin Tissue XS kit (MACHEREY-NAGEL), respectively. Sample quality and DNA concentration were determined via spectrophotometry using an ND-8000 (NanoDrop Technologies, Thermo Fisher Scientific Inc., Wilmington, DE). PCR amplification was conducted using the GeneAmp PCR System 9700 (Applied Biosystems) and the cycling profile was as follows: initial denaturation at 95°C for 5 min, then denaturation, primer annealing, and extension at 95°C for 50 s, 62°C for 50 s and 72°C for 1 min for 35 cycles, followed by a 4 min extension at 72°C. Samples were then cooled and held at 4°C until sequencing. Direct sequencing of the PCR products and processing of raw sequencing data were performed as previously reported in Mereu et al. [17].

### 2.3. Statistical Analysis

The data set encompassed overall 86 *G. fulvus* sequences (Table 1). All the currently available homologous sequences were retrieved from GenBank and included in the analyses performed in the present study. Sequences were aligned using Clustal X2 [23] and BioEdit [24]. Genetic diversity calculations were implemented using DnaSP v5.10 [25]. The number of polymorphic sites (S), number of haplotypes (H), haplotype diversity (h), nucleotide diversity (*π*) and haplotype frequency estimations were calculated as a single population on a 342 bp long fragment encompassing the whole HVS I domain of the mtDNA D-loop region. Calculations of the tests of Fu's Fs and Tajima's D along with the estimation of the coefficient of differentiation (*F_ST_*) and hierarchical analyses of molecular variance (AMOVAs) were performed in ARLEQUIN 3.5.2 [26]. The pairwise genetic distances corrected according to the Kimura two-parameter model (K2P) were estimated between individuals by means of the software MEGA 7.0.14 [27] with 1000 bootstrap replicates. Phylogenetic analysis was performed using Maximum Likelihood (ML) and Bayesian Inference (BI) methods. The phylogenetic trees were rooted by the *G. bengalensis* with a partial 773 bp long sequence from the D-loop (GB# KJ506786) which was aligned to the griffon sequences then trimmed to contain the same regions. The *G. rueppellii,* the closest extant species to *G. fulvus,* was used as additional outgroup. The ML tree was obtained by means of MEGA 7.0.14 [27], which were also used to infer the best nucleotide substitution model. The BI tree was inferred using Mr Bayes 3.2.5 [28] via Markov chain Monte Carlo (MCMC). Samples were drawn every 1,000 steps over 20,000,000 MCMC steps. The first 10% were discarded as burn-in. Acceptable sampling and convergence to the stationary distribution were checked by inspection of traces using Tracerv1.7 [29] and tree topology was edited in FigTree v1.3.1 (http://tree.bio.ed.ac.uk/software/figtree/). In addition, a haplotype network was inferred by means of the Integer Neighbour-Joining option (IntNJ) of PopART v1.7 (http://popart.otago.ac.nz/index.shtml).

Divergence time estimations were calculated using the Bayesian approach implemented in BEAST 1.7.5 [30]. Since no fossil records within *G. fulvus* in-group were available, molecular dating was carried out assuming two calibration points (CPs) inferred by molecular analyses performed by Mereu et al. [17] on the D-loop region sequences. These CPs refer to splitting events within the griffon vulture evolutionary radiation including the *G. fulvus* species appearance (CP1) and the divergence between haplotypes (HPTs) A and B (CP2).

## 3. Results

### 3.1. Genetic Diversity Estimation

Overall, four haplotypes were found among 86 individual griffon vultures; three haplotypes (HPT-A, HPT-B, HPT-C) already described [17] and a new one, previously not reported (HPT-D). The novel haplotype was found only in the Cretan population with a frequency of 6.1%. Differences in these haplotypes were due to four-point mutations including 3 G/A and one C/T transitions. The first polymorphism (G/A) was found at position 37 of the alignment and discriminates HPT-B (nt A) from the other three HPTs (nt G). Two transitions (192: A/G, 301: G/A) were exclusive of HPT-D, while in position 195 a polymorphism C/T discriminates HPTs A and B from HPTs C and D. When considered as one population, haplotype diversity (Hd) was high at 0.474 +/− 0.058, while the nucleotide diversity (*π*) and the average number of nucleotide differences (k) were 0.00176 and 0.606, respectively (Table 2).

The Sardinian and Cretan populations showed a level of genetic diversity characterised by three haplotypes each one (Table 3).

The Sardinian population was composed by 44 sequences (52.4% of the whole sample analysed) distributed among HPTs A (28), B (11) and C (5), with Hd = 0.532 and *π* = 0.00197. The HPT B was found exclusively in sequences from Sardinian griffon individuals. The 33 Cretan sequences (39.3% of the whole sample analysed) were distributed among HPTs A (24), C (7) and D (2), the latter exclusively detected in Cretan specimens, with Hd = 0.436 and *π* = 0.00215. No differences among the 7 Cypriot sequences were found all of them showing the HPT A (Table 3).

The composition, frequency and geographic distribution of each haplotype is reported in Figure 1.

Neutrality tests based on Fu's Fs and Tajima's D were positive and not significant, indicating a nonexpanding population ([Supplementary-material supplementary-material-1]).

An AMOVA, run with each area, resulted in an *F_ST_* of 0.07097 (*p*-value = 0.027 ± 0.014) among the insular populations investigated (Table 4).

The highest level of genetic diversity was observed between HPTs B and D (0.01023 ± 0.00480) which were found exclusives of Sardinian and Cretan populations, respectively, followed by the distance between HPTs A and D (0.00765 ± 0.00427). The lowest divergence was detected when comparing HPT-A to HPTs B and C (0.00254 ± 0.00239) (Table 5).

### 3.2. Phylogenetic Analysis and Molecular Dating

**Figure 2:**
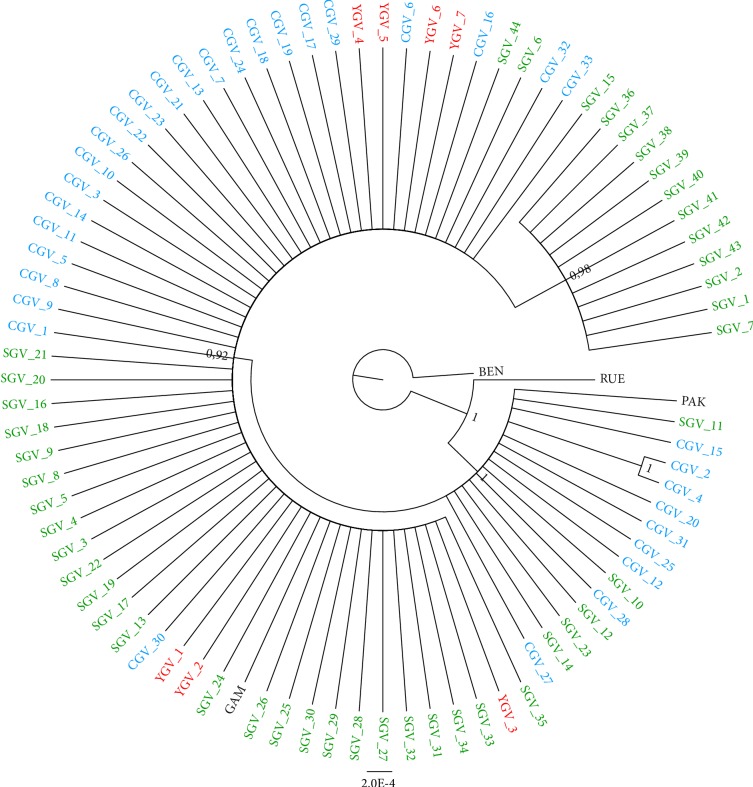
Bayesian rooted tree showing the evolutionary radiation of *G. fulvus* and clarifying the phylogenetic relationships among the four mtDNA haplotypes detected within the entire sample. Statistical support (PP ≥ 0.92) are indicated near each node. SGV: Sardinian griffon vulture; YGV: Cypriot griffon vulture; CGV: Cretan griffon vulture. Sequences from SGV_35 to SGV_15 (clockwise): Hpt A; from SGV_36 to SGV_7: Hpt B; from PAK to CGV_27: HPT-C, excluding CGV_2 and CGV_4 which belong to Hpt D. PAK: sequence from Pakistani individual (DQ908993); GAM: sequence from Gambian individual (DQ908994).

The Bayesian tree analysis supported by high posterior probability (PP) value for each node (PP ≥ 93) is presented in Figure 2. Four different maternal lineages among the whole dataset including 86 griffon vulture sequences were identified. The clades emerging by these lineages correspond to the mtDNA haplotypes reported above. The *G. rueppellii* (RUE) homologous sequence was used as an outgroup, along with the *G. bengalensis* (BEN) sequence that was even used to root the phylogenetic tree. While resulting in similar clustering, ML bootstrap values (data not shown) offered weaker support than BI tree PP to the nodes identifying the main groups retrieved.

**Figure 3:**
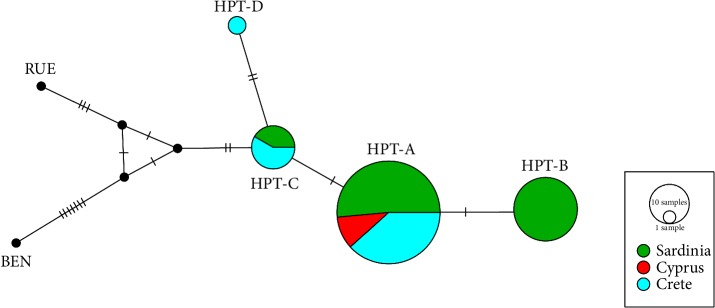
Integer Neighbour Joining network of the four *G. fulvus* haplotypes by geographical area.

The IntNJ network analysis (Figure 3) retrieved four main clusters among the three *G. fulvus *populations analysed in the present study (HPTs A, B, C and D in Figure 3). HPT-A showed the highest frequency both in the entire dataset as well as within each single population and differed for only one polymorphism from HPTs B and C. HPT-D was found to be the rarest one and resulted closely related to HPT-C from which differed for two nucleotide substitutions. HPTs B and D were detected exclusively in the Sardinian and Cretan populations, respectively. Based on our molecular dating, the novel haplotype HPT-D here reported diverged from HPT-C 14,000 years ago (Median values). The rise of the remaining three haplotypes was estimated to have occurred around 780–400 thousand YA.

## 4. Discussion

Evaluating genetic diversity and differentiation of threatened wildlife populations is essential to defining conservation units and developing appropriate conservation and management strategies. In the present study, we analysed three griffon vulture populations from Sardinia, Crete, and Cyprus, in order to estimate the genetic diversity in the *G.fulvus* colonies from these Mediterranean islands. Both Sardinian and Cretan populations showed a genetic structure characterised by three mtDNA haplotypes. No phylogeographic structure has been detected within the entire sample analysed.

Since no mtDNA sequences data from the mainland griffon populations are available for comparative analyses, we cannot infer whether the observed structuring comes from an adaptation to the island environment, successive waves of colonization, bottleneck/genetic drift events or from a mix of all these things. Based on *F_ST_* classes provided by Hartl and Clark [31], we found a moderate level (*F_ST_* = 0.07097) of genetic differentiation among the three griffon populations investigated, according to a limited matrilineal gene flow probably caused by the species reluctance to fly over sea masses or cross long sea stretches and it is confirmed by the analysis on haplotypes frequencies and exclusivity (HPT-D in Crete, HPT-B in Sardinia). This evidence is consistent with Le Gouar et al. [15] which detected high immigration rate in all Continental Europe griffon population and signs of recent isolation due to limited immigration in the Croatian population. A further contribution to the current genetic structure could have come from the well recorded philopatry among female griffons. On the other side, the presence of a single mtDNA lineage in the Cyprus population is probably related to severe bottleneck events although this result should be approached with caution due the limited sample size. HPT-A was found the most common haplotype in all the populations (100% in Cyprus) analysed. However, the prevalence of the HPT A in the Sardinian population was strongly influenced by two restocking actions carried out during the last 30–40 years which modified considerably the haplotype frequencies. Indeed, the HPT B was the most represented haplotype (68.2%) within the pre-restocking population, while the HPT A was found in 31.8% of the specimens [17].

Climatic, geographical and anthropogenic factors should be invoked to explain the observed phylo-geographical pattern of griffon into Mediterranean islands. It is reasonable to suggest two dynamic ways of colonization from mainland griffon populations. The first way could be related to the exploration of new areas by juvenile vultures, which show very high natal dispersion and leave their breeding colonies during the first autumn of life [2, 32, 33]. A second way of dynamic interaction between islands and the mainland is advocated by what happened in Spain in 2008 when a storm moved a group of flying griffons from the mainland to the island of Majorca ca., 170 km away from the Iberian coast [10]. To date, Majorca hosts a healthy griffon colony exhibiting an excellent adaptability to the island's environmental conditions.

We could suppose that at an early stage pioneering individuals lacking social information were attracted by key environmental elements which are fundamental for a griffon colony such as optimal nesting sites and sufficient food availability [34, 35]. Griffon nesting sites need of steep faces characterized by rock formations, with high susceptibility to water erosion that determines a sudden discontinuity in the landscape by shaping habitats not accessible to human activities [36, 37]. Furthermore ascending thermals and orographic air currents deflected upwards by these mountain slopes [38] facilitate taking off of the griffons from the nests. All these environmental elements are typical and abundant in the Mediterranean islands.

Food availability represents another fundamental element to keep griffon colony into an island [39, 35]. The basic food supply of griffons is mostly comprised by medium-large size species of endemic mammalian fauna. Such a food source arrived in the Mediterranean islands during the Neolithic period when the first domestic species, represented by cattle, pigs, goats, and sheep, were transported by the human settlers from the Fertile Crescent through the Mediterranean Sea [40, 41]. Given the vulture species dependence on the big mammal fauna, it is probable that the colonization of the island by griffons was driven from the spread of ruminants towards the Mediterranean islands, which provided a good food availability mainly represented by carcasses of goat and sheep. Both species are bred following an extensive system and constitute the main food source of griffon vultures in the Mediterranean area [22, 42].

## 5. Conclusions

Based on our data, we could suppose that after the first colonization events the Cretan and Sardinian populations remained isolated for a long time during which they maintained genetic variability while the Cypriot colony underwent a drastic bottleneck which only the HPT A survived. However, further analyses including museum specimens should be performed to confirm such a hypothesis.

The different expressiveness of the four mtDNA haplotypes among the three populations analysed in the present study could be explained by assuming that islands colonization occurred in different times. In such a context, each wave of new individuals could have replaced or contributed to enrich the pre-existing gene pool. Most probably, a combination of these two possibilities has determined the genetic variability currently observed in the *G. fulvus *native populations living in the Mediterranean islands. Maintenance of genetic diversity has to be a priority goal for genetic management strategies in long-term conservation programs of the present European griffon. Being places where unique variants not found elsewhere may have been preserved, the islands are often historical memories of past events of which there is no longer any evidence.

Consequently, special attention should be devoted to both these populations, with the aim to facilitate the development and application of appropriate conservation measures. Theory predicts that geographically isolated populations could undergo a progressive reduction of genetic variation causing a decrease of fitness and limiting their ability to face off environmental changes [43]. Gene flow (either natural or artificially aided) between the island and mainland populations could help in maintaining indeed the evolutionary potential of the griffon across Europe and restoring genetic variation within small and endangered natural populations. Further researches based on a multilocus approach investigating on native and reintroduced populations, including those from mainland, would be appropriate to provide a more comprehensive picture of the genetic variability within the Mediterranean griffon populations and significant conservation guidelines in the choice of founder stocks for future reintroduction strategies.

## Figures and Tables

**Table 1:** List of sequences subjected to genetic analyses, showing the number of specimens for each geographic region.

Species	*N*	Geographic origin	Code	GB#
*Gyps fulvus*	44	Sardinia (Italy)	SGV_1–44	KX893248–313
	33	Crete (Greece)	CGV_1–33	MH675929–61^∗^
	7	Cyprus	YGV_1–7	MH675962–68^∗^
	1	Gambia	GAM	DQ908994
	1	Pakistan	PAK	DQ908993

*Gyps rueppellii*	1		RUE	DQ909000
*Gyps bengalensis*	1		BEN	KJ506786

*N*: number; GB#: GenBank accession number; ^∗^Sequences obtained in the present research.

SGV: Sardinian griffon vulture; CGV: Cretan griffon vulture; YGV: Cypriot griffon vulture.

**Table 2:** Genetic diversity estimations within and among the griffon populations investigated in the present study.

Population	*N*	*S*	*h*	*Hd*	*π*	*k*
Sardinian	44	2	3	0.532 (±0.065)	0.00171	0.590
Cretan	33	3	3	0.436 (±0.087)	0.00187	0.644
Cypriot	7	0	1	0.00	0.00	0.00
Tot.	84	4	4	0.474 (±0.058)	0.00176	0.606

*N* = number of sequences; *S* = number of polymorphic sites; *h* = number of haplotypes; *Hd* = haplotype diversity; *π* = nucleotide diversity; *k* = average number of nt differences.

**Table 3:** Haplotype distribution in griffon vulture populations from the Mediterranean islands of Sardinia, Crete and Cyprus.

HPT	Number and percentage of sequences by geographical area			
	Sardinia (%)	Crete (%)	Cyprus (%)	Total *n* (%)
A	28 (63.6)	24 (72.7)	7 (100)	59 (70.2)
B	11 (25)			11 (13.1)
C	5 (11.4)	7 (21.2)		12 (14.3)
D		2 (6.1)		2 (2.4)
Tot.	44 (100)	33 (100)	7 (100)	84 (100)

**Table 4:** AMOVA results with *F_ST_*.

Source of variation	d.f.	Sum of squares	Variance components	Percentage of variation
Among populations	2	1.277	0.01735	7.10
Within populations	81	18.402	0.22718	92.90
Total	83	19.679	0.24453	
Fixation index (*F_ST_*)^∗^		0.07097		

^∗^ *P*-value = 0.03636.

**Table 5:** Pairwise genetic distances (below the diagonal) between haplotypes calculated under the K2P model of nucleotide substitution. Standard error estimates are shown above the diagonal.

	HPT-A	HPT-B	HPT-C	HPT-D
HPT-A		0.00239	0.00244	0.00427
HPT-B	0.00254		0.00342	0.00480
HPT-C	0.00254	0.00509		0.00355
HPT-D	0.00765	0.01023	0.01502	

## Data Availability

The DNA sequences here provided and used to support the findings of this study have been deposited in the National Centre for Biotechnology Information (NCBI) sequence database (https://www.ncbi.nlm.nih.gov/genbank/) under accession numbers GenBank: MH675929–MH675961 for Cretan griffon D-loop sequences and GenBank: MH675962–MH675968 for Cypriot griffon vulture D-loop sequences.

## Supplementary Materials

Supplementary Materials Table S1: Values of neutrality tests (Fs and D) for griffon D-loop region sequences.
