# Supplementary material for: Mitochondrial D-loop Sequence Variability in Three Native Insular Griffon Vulture (Gyps fulvus) Populations from the Mediterranean Basin
Source: Biomed Res Int. 2019 Nov 18;2019:2073919. doi: 10.1155/2019/2073919 (PMC6925705; doi:10.1155/2019/2073919)
Supplement: Supplementary Materials — Table S1: Values of neutrality tests (Fs and D) for griffon D-loop region sequences. [file 2073919.f1.pdf]

TABLE S1: Values of neutrality tests (Fs and D) for griffon D-loop region sequences.

	Statistics	Sardinia	Cyprus	Crete	Mean	S.D.
Tajima's D test						
	Sample size	44	7	33	28.0000	19.0000
	S	2	0	3	1.66667	1.52753
	Pi	1.17970	0.00000	1.28788	0.82253	0.71438
	Tajima's D	2.88407	0.00000	1.70127	1.52845	1.44978
	Tajima's D p-value	0.59350	1.00000	0.34640	0.64663	0.33002
Fu's FS test						
	No. of alleles (unchecked)	3	1	3	2.33333	1.15470
	Theta_pi	1.17970	0.00000	1.28788	0.82253	0.71438
	Exp. no. of alleles	4.85402	0.00000	4.76923	3.20775	2.77832
	FS	2.48727	0.00000	2.45006	1.64577	1.42540
	FS p-value	0.39850	N.A.	0.40270	N.A.	N.A.

S: Number of sites with substitutions

Pi: Mean number of pairwise differences
